# Midwifery

**Published:** 1825-07-01

**Authors:** 


### Midwifery.

1. *Cæsarean Operations.* In a late number of Graefes and Walther's Journal, the following curious case is related by Mr. Ruth, of Upper Silesia :—

Charlotte W——— ætat. 36. who had borne six children, became pregnant a seventh time, according to her own account, about three years and nine months previously to the date of report. She enlarged in size, and thought she could distinctly feel the motions of the fœtus. She became affected with unusual pains in the bowels, that at last were insupportable. She still continued to menstruate. Five or six weeks before the expected delivery, she strained herself, while washing clothes at a river, and felt something give way, at the same time a tumour suddenly formed a little below and on the right side of the navel. She became faint, and was carried home, where she remained confined for five weeks, experiencing a dull pain in the lower part of the abdomen. Labour pains came on, but gradually wore off, leaving the patient in a weak state. After this she resumed her domestic avocations, and continued to do so for upwards of two years, without any particular inconvenience. The menses were regular but scanty. In this situation she again became pregnant, and remained pretty well up to the 7th month, from which period the abdomen became very large and unwieldy, with great failure of strength. She was delivered, however, of a healthy child. Soon after the birth of this child, the mother became very ill, being thirsty, weak, afflicted with diarrhœa and hectic fever, which were rapidly hurrying her to the grave. The abdomen remained large, notwithstanding the general emaciation. There was a small painful tumour below the umbilicus. A surgeon laid this open, conceiving it to be an abscess. A quantity of matter and *hair* came forth. On introducing his finger he felt something hard. It was conjectured to be a fœtus, and it was at this time Mr. Ruth was called in, and found the patient in the situation described. The os uteri was close—the abdomen at the time had the appearance of a person at the full period of pregnancy, and through the opening above-mentioned, the fœtus could be distinctly felt. Mr. R. proposed an operation, which was assented to, and performed on the 31st December, 1822.

*Operation.* An incision was made in the linea alba reaching from above the umbilicus to within a short space of the pubis. The peritoneum was carefully opened, and the abdominal cavity exposed. A fœtus presented itself, and was cautiously removed. The umbilical cord was traced over the uterus to the left side, where it was lost in a softened mass, probably the remains of the placenta. A great quantity of purulent matter was soaked up by a sponge from among the intestines. The uterus appeared larger than natural. The breathing becoming difficult, the wound was sewed up, and the patient put to bed, when an anodyne cordial draught was exhibited. The child was found to be a *female*, 18 or 19 inches in length, and well formed. Many of the external parts were in a state of suppuration—in other respects there was nothing particular in its appearance. The patient remained in a most precarious state for many days; but she gradually recovered, and, by the middle of March she was as well as ever.

*Case* 2. Another case is related in the Medico-chirurgical Gazette of Saltzburg, of which we shall record some particulars in this place. Eliz. H——, 28 years of age, pregnant for the first time. Towards the proper period, she was seized with labour pains, and, in addition, severe convulsions, which continued thirteen or fourteen hours. On attempting to deliver, it was found that the pelvis was not more than two inches wide. Any operation was put off till next morning, as it could not be ascertained whether the child were living or not—*a decision which was to make all the difference between embryotomy and the Cæsarean operation!*—Next morning the woman was found much refreshed, and the motions of the child could be distinctly felt, and *therefore* the Cæsarean section was determined on! An incision was made in the course of the linea alba, in the usual manner, and by another incision the uterus was laid open, while in a state of contraction, from a labour pain. The hand of the operator was introduced, and the placenta detached. The membranes ruptured, the child's body was extracted, but the uterus grasped it round the neck, and it was with some difficulty that the head could be extricated. The placenta was then withdrawn, the uterus cleared of coagula, and the wound brought together by sutures and other proper means. The child was alive—"and made continual efforts to breathe for half an hour," but died at the end of that period. The woman recovered.

*Remarks.* Success is not always a criterion of propriety in surgical operations. We consider the above procedure on the part of the surgeon, as well deserving of condign punishment, whether the woman survived or not. To perform the Cæsarean operation in preference to embryotomy (where the latter is practicable,) is most unwarrantable, and evinces a lamentable, not to say culpable, want of judgment as to the proper estimate of the value of human life.

---

2. *Uterine Tumour.** This case, brought forward by Mr. Lightfoot, is an example of the rapidity and fatality with which fungoid tumours spread their destructive ravages over internal organs.

The patient was a married lady, 56 years of age, who had borne children, and who, till within three years of applying to Mr. L. had enjoyed good health. For the three years mentioned, she had been subject to profuse uterine hæmorrhages, recurring at uncertain periods,

* Mr. Lightfoot. Ed. Journal, Jan. 1825.

and unaccompanied by pain. In the intervals there was considerable mucous discharge from the vagina. This was in March 1815. Some remedies were prescribed, but did no good. In the beginning of April, as pain was complained of in the region of the uterus, and as the system generally was beginning to sympathise with the local affection, an examination was submitted to, and a small but firm tumour was distinctly felt on the posterior side of the *cervix uteri.* Neither it nor the os uteri was very sensible to the touch. Quietude, low living, anodynes, and gentle aperients were prescribed. In the beginning of May, the tumour had greatly increased, and by its pressure on the rectum impeded the discharge of fæces. By the 20th, the tumour nearly filled the hollow of the sacrum; 1st of June, the passage of the fæces was almost entirely prevented. The abdomen became greatly distended and very painful. These symptoms increased so rapidly that death terminated her sufferings on the 9th of the same month.

*Dissection.* The abdomen, which was enormously distended, and irregularly hard, contained a large quantity of serous fluid. The omentum was of scirrhous hardness, and generally adherent to the parts beneath. The viscera were all glued together by adhesive matter. The uterine organs were covered with numerous hydatids,—some of them of large size. The ovaries seemed mere clusters of hydatids. The uterus was double its natural size, with three small tubercles on its anterior surface, two of them spongy, and the other more solid. To the posterior surface of the uterus, towards its cervix, was attached a tumour of immense size, of smooth surface, and with a dense capsule. It descended behind the vagina, and entirely filled the pelvis. When the capsule was cut through, it was found to contain a very vascular fungus that penetrated the posterior surface of the uterus, and had probably given rise to the profuse hæmorrhages from that organ. The intestines were all adherent, and the colon and rectum ready to sphacelate. There were two large hydatids attached to the liver, and its concave surface was studded with other tubera.

"I do not remember ever to have opened a body in which I found so many and such various parasitical productions, hydatids of all sizes, tubercles small and large, spongy and solid, but each retaining its peculiar and specific character; the capsule of the spongy tubercle, however small, secreting, or at least inclosing, a spongy product; the capsule of the solid tubercle a solid; that of the hydatid, however large, a fluid; but I do not recollect to have observed any thing like a conversion of one species of morbid growth into that of another." 75.

Our author concludes by mentioning his success in preventing those formidable hæmorrhages which occasionally follow the expulsion of the placenta, by previous bleeding and antiphlogistic regimen.

---

3. *Rupture of Perinæum.* In No. 8 of the Medical Repository, Mr. Bond has favoured the public with a long paper on this subject and a case in illustration. It is only of the latter we shall present the particulars.

*Case.* "Mrs. ———, aged thirty-two, a delicate woman, of irritable habit, was taken in labour of her first child at half-past two on the morning of the 15th of January. At three o'clock the pains were severe, and the os uteri dilating under their influence. The vagina was so extremely small as to oppose even the introduction of the finger; and I found, upon subsequent inquiry, that even conjugal intercourse had been invariably "quite torture," through the whole nine months of her married life. Notwithstanding this, the head began rapidly to descend as soon as the os uteri had become sufficiently open; the pains became very unusually acute and forcing, and the whole labour was completed before five o'clock. In the interval between two pains the scalp was left protruded between the labia, so as to keep the fourchette upon the stretch; and the next pain extruded the head entirely, tearing down the whole perinæum from the fourchette to the anus. Apprehensive of such an accident from the unusual smallness of the parts, I was assiduous in my endeavours to prevent it; but I need not describe my feelings as I felt the perinæum giving way under my hand during the pain, while all my care was ineffectual to arrest the progress of the laceration. The placenta followed the child immediately. On examining the wound, a separation of its lacerated lips was followed by so profuse a hæmorrhage as to excite great alarm, but it was at once checked by the introduction between them of linen soaked in cold water. I have spoken in a former part of this paper of the position in which the patient was left for the first few hours. At the end of this time, satisfied that only sutures could retain the parts *in situ*, I applied three at proper distances, observing not to draw them more tight than was just necessary to maintain the lips of the wound in apposition, yet endeavouring to secure such hold with each that not only the external edges but the whole of the divided surfaces should be in contact; but in consequence of the looser nature of the integuments close to the anus, the ligature applied in that situation was unavoidably less effectual than either of the other two. On the second day there appeared very severe inflammation and swelling of the parts, producing superficial sloughing of the lower part of the vagina and lips of the vulva. Cold lotions were applied locally, the urine was drawn away twice daily, the bowels were kept open by cooling laxatives, and salines and opiates were administered according to symptoms. The patient was allowed to rest either upon her side or back, moving from one position to the other with great care. I had applied one suture a small distance from the fourchette, with the intention of interposing lint between the lips of the wound in front of it, so that the opening of the vagina might be left somewhat larger than its original size, a second next the anus, and a third between the two. As soon as I considered them of no farther service I removed them. Reunion had taken place along the whole wound, if we except the small portion in front of the ligature nearest the fourchette, and another portion next the anus, in which situation there remained an opening large enough to admit the point of the little finger. It communicated with the vagina, and was, therefore, under existing circumstances, rather a source of gratification than of disappointment, for I was aware that it would fill up in no long time by granulation, and it afforded at present a ready exit for the pus secreted from the interior sloughy surface. After this the patient lay chiefly upon her back; the discharges all passed by this opening, which, however, gradually filled up as their quantity lessened. The vagina was injected with warm water twice daily until the sloughy appearance was no longer visible, and afterwards lint was introduced so as to prevent adhesion between its sides. In short, the whole was entirely healed, and my attendance ceased in less than three weeks from the day of her delivery." 120.

